# Spin-Coated CH_3_NH_3_PbBr_3_ Film Consisting of Micron-Scale Single Crystals Assisted with a Benzophenone Crystallizing Agent and Its Application in Perovskite Light-Emitting Diodes

**DOI:** 10.3390/nano8100787

**Published:** 2018-10-04

**Authors:** Zhan Gao, Yifan Zheng, Dan Zhao, Junsheng Yu

**Affiliations:** State Key Laboratory of Electronic Thin Films and Integrated Devices, School of Optoelectronic Science and Engineering, University of Electronic Science and Technology of China (UESTC), Chengdu 610054, China; 201621050309@std.uestc.edu.cn (Z.G.); yifanzheng_uestc@163.com (Y.Z.); zhaodan@std.uestc.edu.cn (D.Z.)

**Keywords:** single crystal, benzophenone, crystallizing agent, perovskite light-emitting diodes

## Abstract

Owing to the superior properties of optical and electronic properties, perovskite single crystals have been in high demand recently. However, the growth of large-sized single crystals requires several processing steps and a long growth time, which engenders great difficulties in device integration. Herein, benzophenone (BP) was firstly introduced as a crystallizing agent to facilitate the construction of a high-quality CH_3_NH_3_PbBr_3_ (MAPbBr_3_) film consisting of micron-scale single crystals in a one-step spin-coating method. We studied the influence of the BP concentration upon the size and shape of the micron-scale single crystals. Moreover, due to the enhanced morphology of the MAPbBr_3_ film with low-defect micron-scale single crystals, perovskite light-emitting diodes (PeLEDs) have been demonstrated with a maximum luminance of 1057.6 cd/m^2^ and a turn-on voltage as low as 2.25 V. This approach not only proposes a concise and highly repeatable method for the formation of micron-scale perovskite single crystals, but also paves a way for the realization of efficient PeLEDs.

## 1. Introduction

Organic-inorganic hybrid perovskites have been utilized in many optoelectronic applications including perovskite light-emitting diodes (PeLEDs), high-efficiency solar cells, solid-state lasers, and highly-sensitive photodetectors [1,2,3,4,5,6,7,8,9,10] due to their superior photoelectric properties, such as high photoluminescence, tunable luminance, high absorption coefficient, and long carrier lifetime. Although the hybrid perovskite-based optoelectronic devices have been broadly studied, it is worth noting that, in poly-crystalline perovskite materials, defects between boundaries may trap the charges, thus severely depressing the device efficiency. Moreover, the crystallographic defects of poly-crystalline perovskite could also cause the ion migration under electric field, which degrade the stability of the perovskite devices [11,12,13,14,15].

Perovskite single crystals, which exhibit impressive properties: high charge mobility, long carrier lifetime, good crystal stability, and ultra-low trap state densities of the perovskite layers [15,16,17,18,19,20], have great potential to conquer these problems towards high performance devices. In 2015, Shi et al. reported an anti-solvent vapor-assisted crystallization approach to grow crack-free millimeter-sized CH_3_NH_3_PbBr_3_ (MAPbBr_3_) and CH_3_NH_3_PbI_3_ (MAPbI_3_) single crystals. These large single crystals show ultra-low trap state densities on the order of 10^9^ to 10^10^, which is comparable to the best photovoltaic-quality silicon [16]. In 2015, Huang and co-workers created a MAPbI_3_ single crystal by using the top-seeded solution-growth method with a diffusion length in excess of 175 micrometers [17]. Since then, several fabrication technologies, including slow crystallization via the cooling of supersaturated solutions [21], crystallization assisted by the addition of anti-solvent [16], and in specific solvents rapid growth by increasing temperature [22,23], have been subsequently developed. However, most of these crystallization technologies of large-sized single crystals require long growth time and the accurate control of several parameters, such as solution temperature and concentration, causing great difficulties in device fabrication. In addition, most of these previous reports mainly focus on the chemical and optical properties of the single crystal rather than go further to realize applicable devices. In pursuit of high-performance devices, not only should the design of multilayered device architecture be overcome [24], but also the development of novel concise and time-efficient crystallization methods of small-sized single crystals is urgently needed [15,18,25].

Herein, a UV crosslinker named benzophenone (BP) was firstly introduced as a crystallizing agent to facilitate the construction of a high-quality MAPbBr_3_ film consisting of micron-scale single crystals by the spin-coating method. It is found that benzophenone can favor the heterogeneous nucleation of the MAPbBr_3_, lower the energy barrier of nucleation, thus raising the rate of crystallization, making a contribution to the growth of micron-scale MAPbBr_3_ single crystals. Attributed to the enhanced morphology of the MAPbBr_3_ film containing low-defect micron-scale single crystals, its application in PeLEDs was demonstrated with a maximum luminance of 1057.6 cd/m^2^, a maximum current efficiency (CE) of 0.17 cd/A at 7.1 V, and a low turn-on voltage of 2.25 V.

## 2. Experimental 

**Figure 1:**
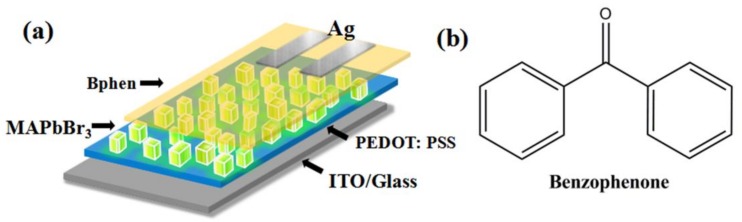
(**a**) Device structure of perovskite light-emitting diodes (PeLEDs); and (**b**) the chemical structure of benzophenone (BP).

Figure 1a describes the structure of the fabricated PeLEDs based on micron-sized MAPbBr_3_ single crystals, which is indium tin oxide (ITO)/poly(3,4-ethylenedioxythiophene):polystyrene sulfonate (PEDOT:PSS) (40 nm)/MAPbBr_3_ (~210 nm)/4,7-diphenyl-1,10-phenanthroline (Bphen) (40 nm)/Ag (~100 nm). Here, we employ PEDOT:PSS and Bphen as a hole transport layer (HTL) and electron transport layer (ETL), respectively. In addition, MAPbBr_3_, ITO, and Ag were used as the emitter, anode, and cathode, respectively. ITO-coated glass substrates with a sheet resistance of 15 Ω/sq were cleaned in an ultrasonic bath with detergent, water, acetone, deionized water, and isopropyl alcohol. Pre-cleaned ITO was treated with oxygen plasma under a pressure of 25 Pa for 5 min after drying in a nitrogen gas flow. PEDOT:PSS was spin-coated at 5000 rpm for 60 s and immediately annealed at 145 °C for 15 min. Then, all the substrates were transferred into a N_2_ glove box. The perovskite precursor solution was prepared by mixing CH_3_NH_3_Br with PbBr_2_ in a molar ratio of 1:1 in *N,N*-dimethylformamide (DMF) with a concentration of 5 wt%. For the growth of MAPbBr_3_ single crystals, benzophenone from Sigma-Aldrich (Shanghai, China) was added in the MAPbBr_3_ solution directly with various concentration from 3 wt% to 6 wt%. The above solution was stirred at 50 °C in air overnight to ensure the adequate dissolution. The perovskite solution was spin-coated onto the PEDOT:PSS film with two steps of spin-coating speed (500 rpm for 10 s and 3000 rpm 120 s). During the spin-coating process, 300 μL of chlorobenzene (CB) was dropped onto the samples at about the 90th second. All the samples were then annealed at 110 °C for 20 min. A 40 nm thick Bphen layer was evaporated to cover the perovskite film, followed by the deposition of Ag (100 nm) through thermal deposition under high vacuum conditions. The overlap between ITO and Ag electrodes was 0.2 cm^2^, which is the active emissive area of PeLEDs. For the fabrication of electron-only devices, the perovskite film is sandwiched by [6,6]-phenyl-C(61)-butyric acid methyl ester (PCBM) layers. PCBM with a concentration of 30 mg/mL was spin-coated on ITO at a rate of 2000 rpm for 60 s inside the glove box. The thickness of PCBM is approximately 40 nm, and Ag (100 nm) is thermally deposited under high-vacuum conditions.

The current density-voltage-luminance (J-V-L) characteristics were tested with a Keithley 4200 source (Beijing, China) and a luminance meter. Both electroluminescence (EL) spectra and Commission International de l’Eclairage (CIE) coordinates of the devices were recorded with a spectrophotometer OPT-2000 (Beijing, China). All the measurements were performed in air at room temperature without encapsulation. Film thickness was measured using a step surface profiler. The surface morphology of perovskite was investigated by scanning electron microscopy (SEM, JEOL JSM-7100F, Chengdu, China). The crystal structure was characterized by X-ray diffraction (XRD, X’Pert PRO, PANalytical, Chengdu, China, Cu Kα radiation λ= 0.154056 nm, 40 kV and 40 mA).

## 3. Results and Discussion

**Figure 2:**
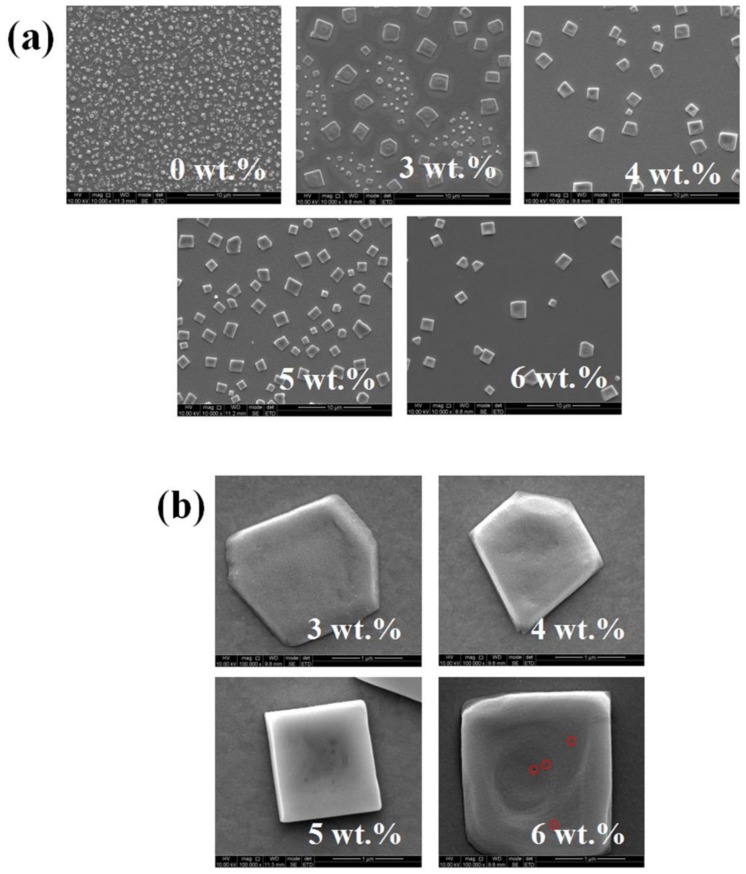
Top-view SEM images of MAPbBr_3_ film. The scale bar is 10 μm in (**a**) and 1 μm in (**b**).

The MAPbBr_3_ film consisting of micron-scale single crystals was fabricated by introducing BP as a crystallizing agent in a one-step spin-coating method. To investigate the effects of BP upon the morphology of the perovskite film, the top-view SEM is employed. The images of the MAPbBr_3_ film with different concentrations of BP are shown in Figure 2a,b. It can be seen that without BP, the MAPbBr_3_ film has rather poor morphology with round poly-crystalline grains which are both nonuniform in shape and size, as shown in our previous works [26]. This poor morphology and high density of defects would lead to significant non-radiative loss of devices. In contrast, when doping BP in the MAPbBr_3_ solution, the film morphology and perovskite crystallinity are greatly enhanced. With a low BP concentration of 3 wt%, the growth of micron-scale single crystals are not yet complete. The film shows irregular cubic-shape grains (hexagonal-shaped) which are surrounded by smaller grains. As the BP concentration increases to 4 wt%, the smaller grains disappear, while large grains show a more cubic-shaped. This enlarged size of grains may result from the lower energy barrier of the nucleation of PbBr_2_ induced by BP [27]. BP may favor the heterogeneous nucleation of the MAPbBr_3_, reducing the energy fluctuation and structure fluctuation of the nucleation and the rate at which crystallization rises, which contributes to the growth of micron-scale MAPbBr_3_ single crystals [27,28,29,30,31]. For the optimized BP concentration with 5 wt%, the film shows almost perfect cubic-shaped grains, indicating the highly crystalline and fewer defects of micron-sized single crystals. As the concentration further increase to 6 wt%, the grains are sparse and the size of crystals become uneven in that the largest size of the crystals exceeds 2 µm. Too large grains fail to confine charges and the probability of radiation recombination decreases, leading to a decline of device performance [15]. Moreover, the geometry of micron-sized crystals deteriorates to a sunken surface with some pinholes (red circle in Figure 2b).

**Figure 3:**
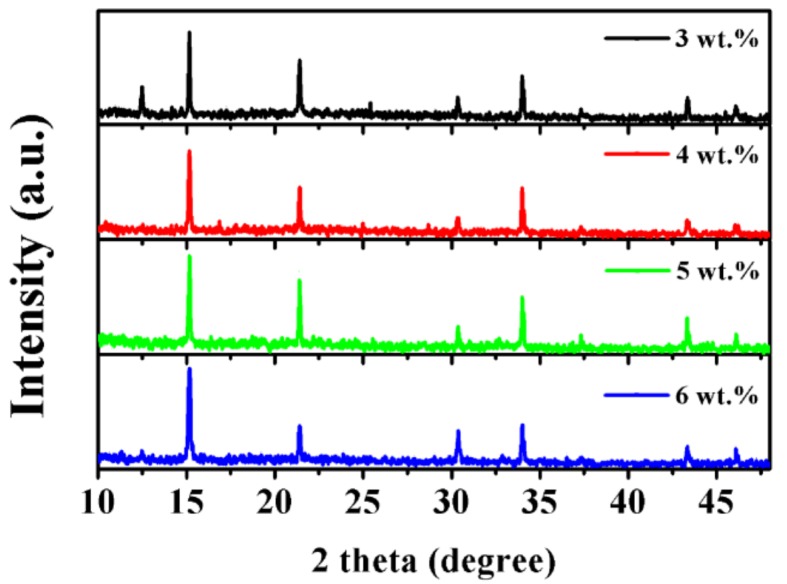
X-ray diffraction patterns of the MAPbBr_3_ film.

The X-ray diffraction patterns (XRD) of the MAPbBr_3_ film are shown in Figure 3. In contrast to the other BP concentrations, 3 wt% BP-doped MAPbBr_3_ film shows the appearance of a diffraction peak at 12.5°, indicating the undesired δ-phase, which is consistent with the hexagonal-shaped single crystal observed in Figure 2b. The δ-phase may induce serious PeLED performance drop [32] resulting from the unstable phase of perovskite, which may suppress light emitting efficiency. As the BP concentration increases, the δ-phase of MAPbBr_3_ has been successfully inhibited and exhibited a characteristic cubic structure fingerprint ($Pm\bar{3}m$) [33]. For the optimized 5 wt% BP-assisted MAPbBr_3_ film, the diffraction peaks are shown at 15.1°, 21.4°, 30.3°, 33.9°, 43.3°, and 46.2°, corresponding to the (100), (110), (200), (210), (220), and (300) lattice planes. This result is in good agreement with the previous works [16,19]. The UV–VIS absorption and photoluminescence (PL) spectra for the films with different BP are shown in Figures S1 and S2, respectively. The overlapped absorption features of perovskite films with each BP concentration have an absorption band of 531 nm. Meanwhile, no obvious changes can be observed from the PL spectra with each BP concentration.

**Figure 4:**
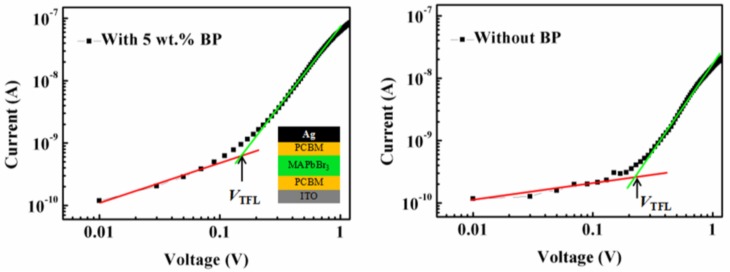
I–V characteristic of an electron-only device based on MAPbBr_3_ film, with an inset showing the device structure.

To unravel the charge recombination loss in the perovskite film, the electron-only devices (ITO/PCBM/MAPbBr_3_/PCBM/Ag) were fabricated to study the trap density by using the space charge limited current (SCLC) method [16,20,34,35]. The I–V curves of the devices with and without the assistance of the BP crystallizing agent are shown in Figure 4. The linear I–V relation (red line) indicates an ohmic response at low bias, and the current increase nonlinearly when the bias voltage exceeds the trap-filled limit voltage (*V*_TFL_), demonstrating that all the available trap states are filled by the injected carriers. The onset voltage *V*_TFL_ is linearly proportional to the density of trap states *η*_t_, which follows the equation:

*V_TFL_* = *eη_t_L^2^/2εε_0_* (1)

where *e* is the elementary charge of the electron (*e* = 1.6 × 10^−19^ C), *L* is the perovskite film thickness (≈210 nm), *ε* is the relative dielectric constant of MAPbBr_3_ (here we use 25.5 [16]), *ε*_0_ is the vacuum permittivity (*ε*_0_ = 8.854 × 10^−12^ F/m), and *η*_t_ is the trap state density of MAPbBr_3_ film. The *V*_TFL_ of the MAPbBr_3_ film with and without BP can be identified in Figure 4, respectively. The thickness of the perovskite film with and without BP is ~80 nm and 210 nm, respectively, which can be obtained from the cross-SEM image and the statistical results of the film thickness as shown in Figures S3 and S4. For the non-uniform nature of the film, these calculations of the trap density of electron are estimations. The electron trap state density of MAPbBr_3_ film with BP is 8.9 × 10^15^ cm^−3^, which is two orders of magnitude lower than the MAPbBr_3_ film without BP (1.3 × 10^17^ cm^−3^). This decrease of trap density derives from the high-quality and enhanced morphology of the MAPbBr_3_ film with micron-scale single crystals. It can benefit the transportation of charge carriers, reduce non-radiative loss and, thus, promote the performance of PeLEDs accordingly.

**Figure 5:**
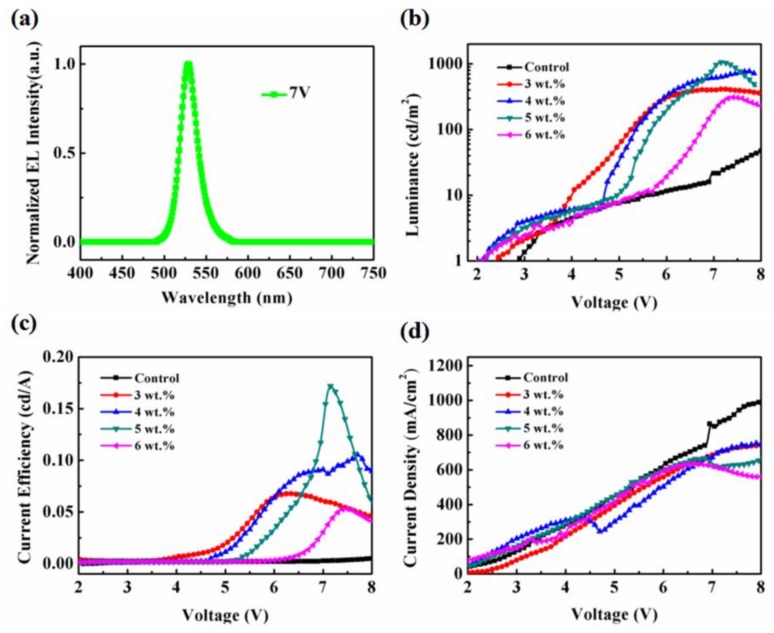
(**a**) Normalized EL spectra of PeLEDs at 7 V. (**b**) Luminance versus voltage curves of PeLEDs. (**c**) Current efficiency versus voltage curves of PeLEDs. (**d**) Current density versus voltage curves of PeLEDs.

**Figure 6:**
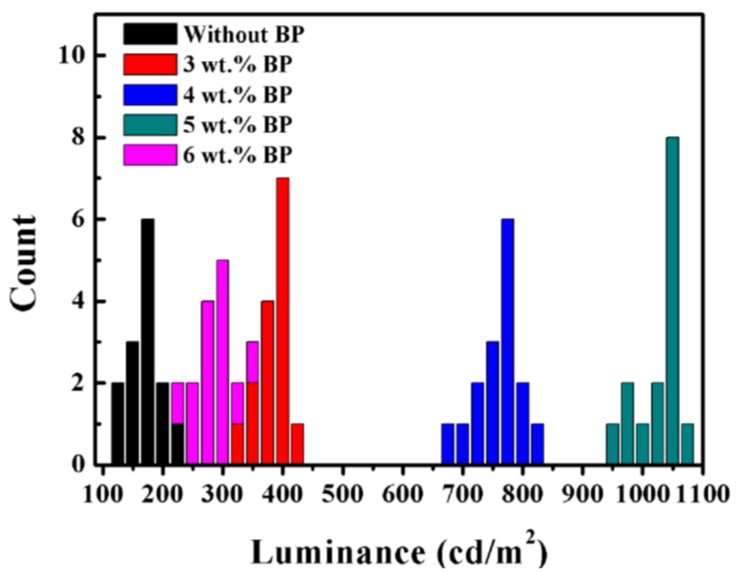
Performance distribution of PeLEDs with each BP concentration.

Finally, the MAPbBr_3_ film comprising of micron-scale single crystals is used to fabricate PeLEDs, and the properties of luminance, device current efficiency, and voltage-dependent current density are shown in Figure 5. As shown in Figure 5a, the EL spectra of the BP modified devices are tested, which is located peaking at 531 nm with a full width at half maximum of 22 nm, corresponding to the calculated CIE coordinates of (0.18, 0.77). It should be pointed out that no additional emission peaks appear in the EL spectra, suggesting that BP has no influence on the high color purity of the device. The performance of PeLEDs based on the MAPbBr_3_ film consisting of micron-scale single crystals is significantly enhanced, compared to the control device based on poly-crystalline MAPbBr_3_. It can also be seen in Figure 5b,c that, due to the poor morphology and high density of defects, the control PeLED without the BP crystallizing agent exhibits a modest performance with a maximum luminance of 171.7 cd/m^2^, and a maximum CE of 0.015 cd/A at 10.3 V. When a low concentration of BP with 3 wt% in perovskite solution is introduced, the maximum luminance increases slightly up to 411.1 cd/m^2^ and the maximum CE reaches up to 0.067 cd/A, respectively. As the BP concentration increases to 4 wt%, the devices show better performance with 775.9 cd/m^2^ and a maximum CE of 0.105 cd/A at a bias of 7.7 V. The optimized devices with 5 wt% BP show the highest performance of a maximum luminance of 1057.6 cd/m^2^ and maximum CE of 0.17 cd/A at a bias of 7.1 V. We attribute this great improvement of device performance to the improved morphology and high quality of the MAPbBr_3_ film with micron-scale single crystals, which can make full use of charge carriers injected in the emissive layer. Moreover, the devices based on micron-scale single crystals show lower turn-on voltage in Table 1 and current density than the control device by fabrication of low-defect micron-scale single crystals, which suppress trap-assisted nonradiative loss [15]. As a result, it significantly reduces the leakage current and loss of non-radiative recombination, leading to a higher luminance and efficiency of PeLEDs. However, when the BP concentration increases to 6 wt%, the device performance drops dramatically. This could be ascribed to the large grain size and pinholes on the surface of the micron-scale single crystals, leading to the inefficient confine of the charge carriers within grains and undesired non-radiative loss. In addition, 75 devices based on each concentration of BP were fabricated through the same fabrication process, and their maximum luminances were tested, as shown in Figure 6. It can be seen that the device performance is very reproducible, indicating that the devices based on the MAPbBr_3_ film consisting of micron-scale single crystals possess the improved luminance.

## 4. Conclusions

In summary, a high-quality MAPbBr_3_ film consisting of micron-scale single crystals has been realized by introducing a crystallizing agent of BP. The result showed that BP can favor the heterogeneous nucleation of the MAPbBr_3_ and lower the energy barrier of nucleation, thus facilitating the growth of micron-scale MAPbBr_3_ single crystals. The morphology of the MAPbBr_3_ film is closely related to the BP concentration and, with an optimized BP concentration of 5 wt%, the best morphology and crystallization of the MAPbBr_3_ film was obtained. Then the PeLEDs were fabricated based on the obtained MAPbBr_3_ film, and the enhanced morphology of the MAPbBr_3_ film comprising of low-defect micron-scale single crystals can suppress the current leakage and the loss of non-radiative recombination. In contrast to the control device, the PeLEDs processed from 5 wt% BP solution present a significant performance enhancement with a maximum luminance of 1057.6 cd/m^2^ at 7.1 V and a lower turn-on voltage of 2.25 V. This work develops a concise and time-efficient method to process a high-quality MAPbBr_3_ film composed of micron-scale single crystals, and opens a universal route for the future realization of high-performance PeLEDs.

## Figures and Tables

**Table 1:** Summarized performance of PeLEDs without and with the BP crystallizing agent.

Concentration	Luminance_max_ (cd/m^2^)	CE_max_ (cd/A)	Turn-on Voltage (V)
0 wt% BP	171.1 @ 10.3 V	0.015 @ 10.3 V	2.95 V
3 wt% BP	411.1 @ 7.1 V	0.067 @ 6.3 V	2.40 V
4 wt% BP	775.9 @ 7.7 V	0.105 @ 7.7 V	2.20 V
5 wt% BP	1057.6 @ 7.1 V	0.171 @ 7.1 V	2.25 V
6 wt% BP	310.4 @ 7.4 V	0.053 @ 7.4 V	2.35 V

## Supplementary Materials

The following are available online at http://www.mdpi.com/2079-4991/8/10/787/s1, **Figure S1.** UV–VIS absorption of MAPbBr3 films with each BP concentration; **Figure S2.** PL spectra of the films with each BP concentration; **Figure S3.** Cross-SEM of the perovskite films: (**a**) CH_3_NH_3_PbBr_3_ film consisting of micron-scale single crystals; and (**b**) poly-crystalline CH_3_NH_3_PbBr_3_ film. **Figure S4.** The statistical results of the thickness of the films: (a) the perovskite film without BP; and (b) the perovskite film with 5 wt% BP.
