# Supplementary material for: Spin-Coated CH3NH3PbBr3 Film Consisting of Micron-Scale Single Crystals Assisted with a Benzophenone Crystallizing Agent and Its Application in Perovskite Light-Emitting Diodes
Source: Nanomaterials (Basel). 2018 Oct 4;8(10):787. doi: 10.3390/nano8100787 (PMC6215164; doi:10.3390/nano8100787)
Supplement: Supplementary file 1 [file nanomaterials-08-00787-s001.pdf]

Spin-Coated $CH_3NH_3PbBr_3$ Film Consisting of Micron-Scale Single Crystals Assisted with a Benzophenone Crystallizing Agent and Its Application in Perovskite Light-Emitting Diodes

Zhan Gao, Yifan Zheng, Dan Zhao, Junsheng Yu*

State Key Laboratory of Electronic Thin Films and Integrated Devices, School of Optoelectronic Science and Engineering, University of Electronic Science and Technology of China (UESTC), Chengdu 610054, PR;

* Corresponding author.
E-mail addresses: jsyu@uestc.edu.cn (J.Yu).

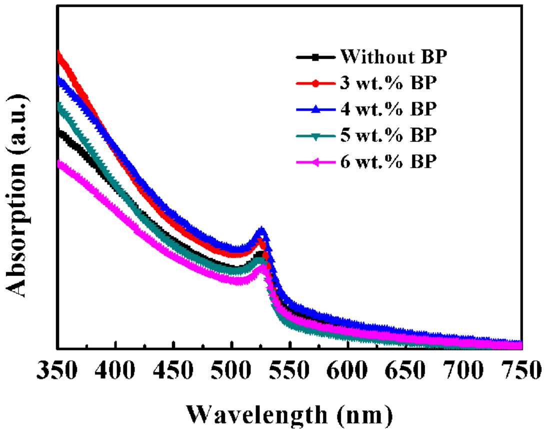

Figure S1. UV－VIS absorption of $MAPbBr_3$ films with each BP concentration.

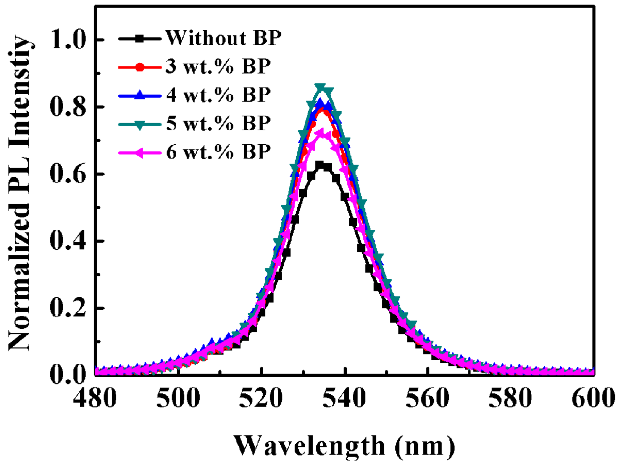

Figure S2. PL spectra of perovskite films with each BP concentration.

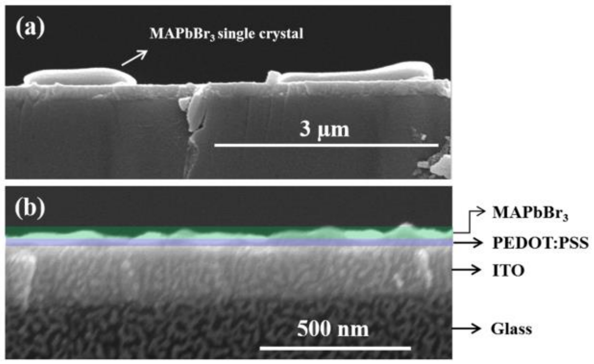

Figure S3. Cross-SEM of $CH_3NH_3PbBr_3$ films. (**a**) $CH_3NH_3PbBr_3$ film consisting of micron-scales single crystals; and (**b**) poly-crystalline $CH_3NH_3PbBr_3$ film.

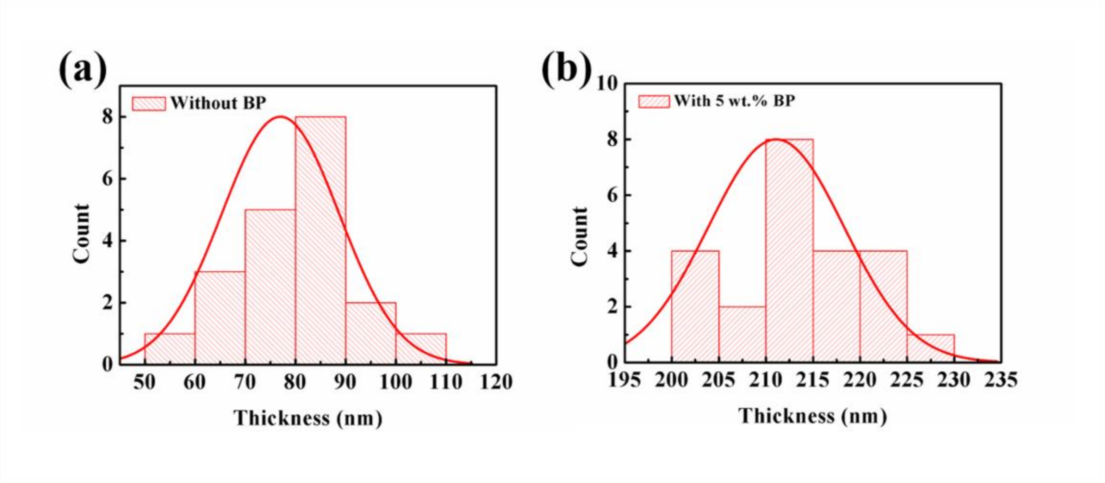

Figure S4. The statistical results of the thickness of the films: (**a**) the perovskite film without BP; and (**b**) the perovskite film with 5 wt% BP.
